# Functional Implications of the Dynamic Regulation of EpCAM during Epithelial-to-Mesenchymal Transition

**DOI:** 10.3390/biom11070956

**Published:** 2021-06-29

**Authors:** Taylor C. Brown, Narendra V. Sankpal, William E. Gillanders

**Affiliations:** 1 Department of Surgery, Washington University School of Medicine, St. Louis, MO 63110, USA; sankpaln@wustl.edu (N.V.S.); gillandersw@wustl.edu (W.E.G.); 2 Siteman Cancer Center, Washington University School of Medicine, St. Louis, MO 63110, USA

**Keywords:** epithelial cell adhesion molecule (EpCAM), epithelial cancers, epithelial-to-mesenchymal transition (EMT), metastasis, circulating tumor cells (CTCs), cancer stem cells (CSCs)

## Abstract

Epithelial cell adhesion molecule (EpCAM) is a transmembrane glycoprotein expressed in epithelial tissues. EpCAM forms intercellular, homophilic adhesions, modulates epithelial junctional protein complex formation, and promotes epithelial tissue homeostasis. EpCAM is a target of molecular therapies and plays a prominent role in tumor biology. In this review, we focus on the dynamic regulation of EpCAM expression during epithelial-to-mesenchymal transition (EMT) and the functional implications of EpCAM expression on the regulation of EMT. EpCAM is frequently and highly expressed in epithelial cancers, while silenced in mesenchymal cancers. During EMT, EpCAM expression is downregulated by extracellular signal-regulated kinases (ERK) and EMT transcription factors, as well as by regulated intramembrane proteolysis (RIP). The functional impact of EpCAM expression on tumor biology is frequently dependent on the cancer type and predominant oncogenic signaling pathways, suggesting that the role of EpCAM in tumor biology and EMT is multifunctional. Membrane EpCAM is cleaved in cancers and its intracellular domain (EpICD) is transported into the nucleus and binds β-catenin, FHL2, and LEF1. This stimulates gene transcription that promotes growth, cancer stem cell properties, and EMT. EpCAM is also regulated by epidermal growth factor receptor (EGFR) signaling and the EpCAM ectoderm (EpEX) is an EGFR ligand that affects EMT. EpCAM is expressed on circulating tumor and cancer stem cells undergoing EMT and modulates metastases and cancer treatment responses. Future research exploring EpCAM’s role in EMT may reveal additional therapeutic opportunities.

## 1. Introduction

Epithelial cell adhesion molecule (EpCAM) is an approximately 40 kilodalton (kD), type I transmembrane glycoprotein. The human *EPCAM* gene is located on chromosome 2 (2p21) and has an estimated size of 14 kilobases (kb) [1]. EpCAM is expressed on the basolateral membrane of most epithelial tissues and the highest expression levels are observed in colonocytes. Limited expression is observed in some epithelial types (e.g., squamous cells of the skin and hepatocytes) and mesenchymal cells, including nervous, connective, and bone-marrow-derived cells [2,3]. EpCAM plays a prominent role in forming intercellular adhesions, maintaining the epithelial integrity of the gastrointestinal tract, and promoting the epithelial phenotype across various tissue types.

Embryological studies demonstrate that EpCAM plays an important role in epithelial organ development and placental formation [4]. Germline EpCAM mutations cause congenital tufting enteropathy (CTE), a chronic diarrheal illness that typically presents in neonates and causes intractable diarrhea and intestinal failure [5]. Mutations associated with CTE prevent surface expression of EpCAM [6], which in turn causes villous atrophy, the formation of epithelial tufts [7], and the aberrant expression of cellular adhesion proteins including integrins, desmogleins, E-cadherin, β-catenin, claudin-7, occludin, and zona occludens-1 [8,9,10,11]. This results in intestinal barrier and ion transport dysfunction and diarrheal illness [10,11]. In addition to its adhesive role, EpCAM can also function as a signaling molecule. Upon cleavage of full length EpCAM at the cell membrane, EpCAM’s intracellular domain (EpICD) serves as a nuclear signaling molecule along with β-catenin and regulates the transcription of target genes [12]. EpCAM’s extracellular domain (EpEX) can also be cleaved and secreted, and recent studies have shown it can serve as a ligand for the epidermal growth factor receptor (EGFR). EpCAM’s precise role in EGFR signaling remains to be fully clarified [13].

In addition to its role in healthy tissues, EpCAM plays a prominent role in cancer biology and was the first human tumor-associated antigen to be identified using monoclonal antibodies [14]. Subsequent studies have shown that EpCAM is frequently and strongly expressed on the cell surface of many epithelial cancer types, with the highest expression levels observed in adenocarcinomas of the pancreas, colon, and prostate [15]. Due to its high antigenicity and expression, EpCAM has been a target of monoclonal antibody therapies in colorectal cancer [16,17]. More recently, the trifunctional antibody Catumaxomab, which targets EpCAM on epithelial cells and mitigates a multipronged, anti-cancer immune response [18], has been used in the treatment of malignant ascites in epithelial cancers. Clinical trials have demonstrated promising results [19,20,21] and Catumaxomab has been approved for the intraperitoneal treatment of patients with malignant ascites in Europe [18]. EpCAM is also frequently the target of methods to capture and measure circulating tumor cells (CTCs) in epithelial cancer patients [22], which provides important prognostic information and can help to determine the response to therapeutic interventions.

During the last three decades, an expanding body of research has demonstrated that EpCAM has many versatile functions that have important implications for cancer initiation, progression, and the underlining mechanisms of epithelial-to-mesenchymal transition (EMT). EMT is a differentiation program typically observed in higher order species, including vertebrates, that allows epithelialized cells to differentiate into mesenchymal cells. EMT enables cell migration in the developing embryo and is essential for organogenesis and tissue regeneration [23]. In cancer, EMT is a complex process in which epithelial cancer cells alter key characteristics and assume a mesenchymal phenotype that enables cell motility, invasion, and metastasis. Broadly speaking, there are several hallmark modifications that occur during EMT. First, epithelial cells deconstruct their intercellular junctions, including tight junctions, adherens junctions, desmosomes, and gap junctions. In turn, epithelial cells lose their apical-basal polarity and acquire a front-rear organization. Second, cytoskeleton actin is reorganized to form actin stress fibers and cellular extensions, including lamellipodia, filopodia, and invadopodia, which enables directional migration. Finally, cells gain the ability to degrade extracellular matrix (ECM) proteins, which promotes invasive behavior. These changes are mediated by complex signaling cascades and transcription factor activity [24].

During many of the aforementioned processes, EpCAM is increasingly being demonstrated to play an important role in EMT. EpCAM is typically downregulated during EMT in epithelial cancers. This is mediated by extracellular signal-regulated kinases (ERK), EMT transcription factor activity, and other oncogenic mechanisms and pathways. During EMT, EpCAM can also be cleaved during a process called regulated intramembrane proteolysis (RIP) and its intracellular domain (EpICD) is transported into the nucleus and helps to stimulate target gene transcription that promotes growth, cancer stem cell properties, and EMT. EpCAM also appears to have a functional impact on EMT. Given the multifunctional role of EpCAM, modulation of EpCAM expression can have differential outcomes on tumor biology and EMT and EpCAM can either inhibit or promote EMT. These observations are typically context dependent and are based on the cancer type that is being analyzed. As discussed below, EpCAM typically promotes malignant properties in epithelial cancers, but can have an inhibitory or negligible impact in mesenchymal cancers. In addition, EpCAM’s different protein domains have unique roles in EMT and their function is also dependent on the cellular and/or extracellular location of their expression. In some studies, it is not clear which domain of EpCAM is causing the observed effect on EMT. Our expanding knowledge of EpCAM’s role in EMT has provided important pathological markers for cancer patients and is revealing potential therapeutic opportunities.

## 2. EpCAM Forms Intercellular Adhesions and Promotes Cellular Segregation and Aggregation

**Figure 1:**
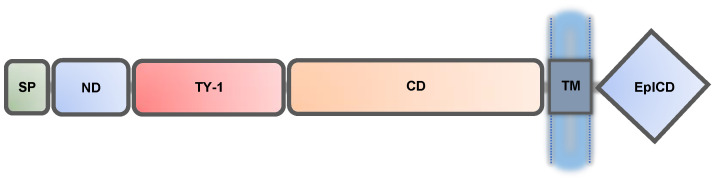
EpCAM has six domains. The protein consists of 314 amino acids. The signal peptide (SP) is typically cleaved, but is shown here. This is followed by the N-terminal domain (ND), the type-1 thyroglobulin domain (TY-1), and the carboxyl-terminal domain (CD). EpCAM has a single pass transmembrane domain (TM) and an intracellular domain (EpICD).

EpCAM is a unique cell adhesion molecule (CAM) that does not resemble other classical adhesion molecules (e.g., cadherins, selectins, integrins, and immunoglobulin-like-CAMs) in either structure or function. EpCAM’s protein structure (Figure 1) is highly conserved across different species, especially in higher vertebrates including humans [1]. EpCAM consists of a large extracellular component (242 amino acids) with a smaller single-pass transmembrane (23 amino acids) and an intracellular (26 amino acids) structure. EpCAM has 314 amino acids and six described domains: signal peptide (SP), N-terminal domain (ND), type-1 thyroglobulin domain (TY-1), carboxyl-terminal domain (CD), transmembrane domain (TM), and intracellular domain (EpICD). Recent structural studies demonstrate that EpCAM’s extracellular tertiary structure assumes a heart, or triangle shape and EpCAM frequently forms *cis* dimers on epithelial cell surfaces [25,26].

Early functional studies demonstrated that EpCAM forms physical interactions between cells, functions as a cell adhesion molecule, and causes cell aggregation. In a seminal study published in 1994, Litvinov et al. force-expressed EpCAM in murine fibroblast and breast cancer cell lines and demonstrated that EpCAM forms homophilic, intercellular adhesions. This in turn caused cell aggregation and cell sorting/segregation from non-EpCAM expressing cells. Unlike other classical CAMs (e.g., cadherins), this occurred in a calcium independent manner, which is a unique feature of EpCAM. It is also important to note that the adhesions formed by EpCAM were weaker than those formed by E-cadherin [27]. These findings were also supported by experiments in human tissues and human breast and ovarian cancer cell lines. EpCAM was shown to localize predominantly along the lateral membrane at points of contact in polarized cells, but did not appear to interact with extracellular matrix proteins including fibronectin, collagens, and laminin. A neutralizing antibody that targeted the extracellular domain of EpCAM was able to abrogate EpCAM’s adhesive properties [28]. Using immunofluorescent and electron microscopy techniques, EpCAM was also shown to form intercellular adhesion structures in the proximity of, but separate from, cadherins. EpCAM was not present near tight junctions or desmosomes [29]. It was further demonstrated that EpCAM’s ability to form intercellular adhesions and affect cellular aggregation was dependent on EpCAM’s binding of α-actin by its cytoplasmic tail. Cytoplasmic tail mutations reduced EpCAM’s localization to the cell surface and decreased EpCAM’s aggregative abilities [30].

Other studies have elucidated the structural organization of intercellular adhesions formed by EpCAM. Chemical crosslinking and EpCAM crystal structure studies demonstrated that EpCAM *cis* dimers likely align in a head-to-head orientation and form a rhombohedral-shaped trans-tetramer that mediates cell adhesions [25,31]. Balzar et al. proposed a different model with the formation of *trans* octamers from *cis* tetramers. They also demonstrated that EpCAM mutations in the extracellular domain and EpCAM antibodies could abrogate both *trans*-cellular and *cis*-cellular interactions between EpCAM proteins [32].

Some studies demonstrate, however, that EpCAM may not form intercellular adhesions. Gaber et al. recently reported their findings of multiple experimental assays that did not demonstrate EpCAM forming intercellular adhesions. EpEX oligomer formation in solution was analyzed using small angle X-ray scattering (SAXS) and tetramers that would form intercellular adhesions were not detected. Chemical cross-linking coupled with mass spectroscopy identified the position of EpCAM oligomer interactions. However, based on a detailed computational analysis, the detected interactions were not predicted to support the formation of *trans* tetrameric adhesion units. The investigators also determined that EpCAM *cis* dimers did not form *trans* interactions using bead aggregation assays (BAA) and fluorescence-lifetime imaging based Förster resonance energy transfer (FILM-FRET) [26]. Fagotto et al. recently provided a critical review of earlier experimental data and questioned EpCAM’s ability to affect transcellular adhesions. In particular, the authors noted that the small and sparsely disbursed EpCAM adhesions identified by electron microscopy would not be capable of causing the broad and close approximation of the cell membrane that was being observed [33]. While these two reports provide important evidence and critical questions, further investigation is needed for clarification. Most studies, however, show that EpCAM is a CAM that forms homophilic, intercellular adhesions, maintains epithelial integrity, and promotes cellular aggregation.

## 3. Transcriptional Control of EpCAM Expression and its Downregulation during EMT

**Figure 2:**
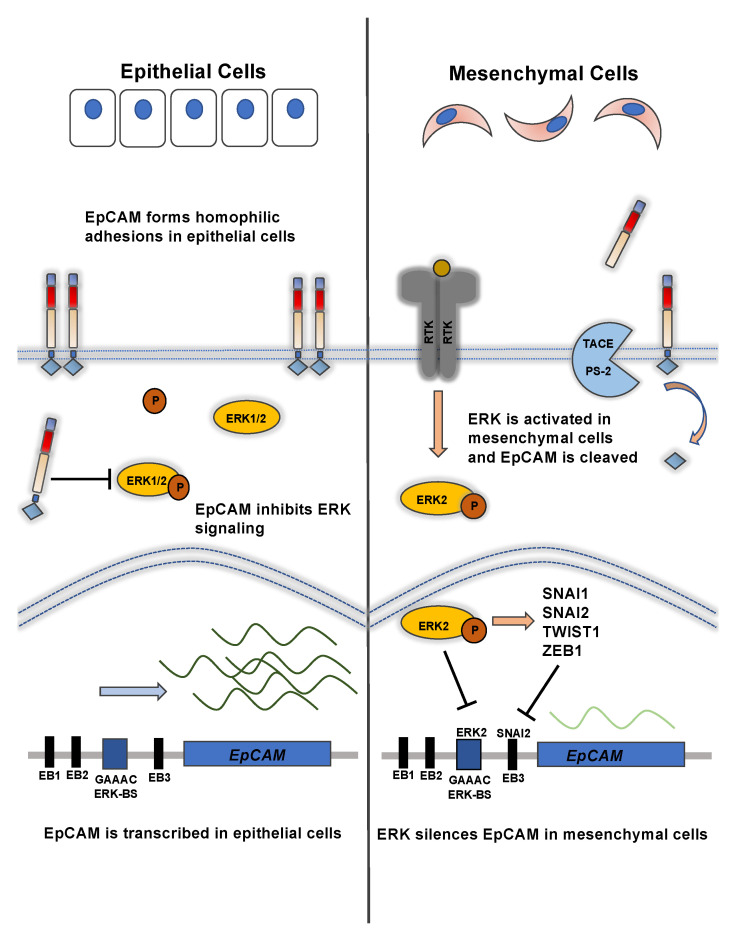
The regulation of EpCAM during EMT occurs through a double-negative feedback loop. In epithelial cells (left panel), EpCAM forms homophilic adhesions and promotes epithelial homeostasis. EpCAM can also inhibit ERK signaling (a potent mediator of EMT) and its gene targets, including SNAI2, EGR1, FOS, JUN, ATF3, and others. In mesenchymal cells (right panel), ERK pathway signaling silences EpCAM expression. ERK2 binds the EpCAM promoter at an ERK2-binding consensus sequence and directly inhibits EpCAM gene expression. ERK2 also induces EMT transcription factors, including SNAI2, that in turn also inhibit EpCAM transcription.

As previously mentioned, EpCAM is expressed in epithelial organs, while limited expression is observed in mesenchymal tissues [2,3]. A similar pattern has been extensively characterized in epithelial and mesenchymal cancers [34] and will be discussed in greater detail below. In a comprehensive analysis, EpCAM gene silencing was identified to be part of a 76 gene signature of EMT [35]. EpCAM expression is highly regulated by transcriptional control mechanisms (Figure 2). Many different signaling proteins and transcription factors, including those that have prominent roles in cancer initiation and progression and EMT, have been shown to either directly or indirectly silence EpCAM promoter activity and expression during EMT.

In an important study, Sankpal et al. demonstrated that EpCAM is dynamically regulated during EMT in a novel double-negative feedback loop in epithelial cancers. Double-negative feedback loops promote bi-stable states, whereby the products of one state (e.g., proteins) prevent the activation of the alternative state and vice versa. Double-negative feedback loops are frequently observed in biological systems [36]. In silico and in vitro analysis demonstrated that EpCAM silencing is associated with increased ERK pathway activation, which is a potent mediator of EMT. ERK2 was shown to bind the EpCAM promoter at an ERK2-binding consensus sequence and directly inhibit EpCAM gene transcription. ERK2 also induced EMT transcription factor activity (SNAI1, SNAI2, and TWIST1), which in turn also inhibited EpCAM gene transcription. Surprisingly, EpCAM was able to inhibit ERK signaling and ERK gene targets in EMT, including SNAI2, EGR1, FOS, JUN, ATF3, and others in MCF-10A breast epithelial cells, thus forming a double-negative feedback loop [37]. The precise mechanism by which EpCAM inhibits ERK activity remains to be clarified. In contrast, in a mouse model of liver fibrosis, increased ERK activation was associated with cell proliferation and EpCAM overexpression, though it was not specifically tested if ERK activation caused EpCAM overexpression [38]. Thus, the role of ERK signaling on EpCAM expression may also be dependent on the tissue type.

ZEB1 is an E-box transcriptional repressor that induces EMT. In lung, breast, and pancreatic cancers, ZEB1 was shown to either directly or indirectly inhibit transcription of EpCAM. This was facilitated by ZEB1 binding of the EpCAM promoter and by ZEB1 induced changes in histone acetylation levels [39,40,41]. Similarly, EMT transcription factors SNAI1, SNAI2, and TWIST1 have been shown to negatively regulate EpCAM transcription in epithelial cancers [37]. In a study examining EpCAM’s regulation in ovarian cancer cell lines, van der Gun et al. identified multiple mechanisms that modulated EpCAM expression, including transcription factor signaling [42]. Several of these transcription factors, including ETS1, LEF1, NF-κβ, SP1, and STAT3, have been implicated in promoting EMT [43,44,45,46,47], though their exact impact on EpCAM gene expression remains to be fully clarified. Wnt-β-catenin signaling, which is frequently upregulated in cancer and promotes EMT [48], can stimulate EpCAM expression. Yamashita et al. demonstrated that a transcription factor 4 (TCF4)/β-catenin protein complex binds Tcf binding elements in the EpCAM promoter and promotes EpCAM expression and in vitro growth in hepatocyte and hepatocarcinoma cell lines [49].

Additional studies have shown that epigenetics changes are an important regulator of EpCAM expression. In ovarian cancer, gene promoter methylation and histone modifications were associated with EpCAM expression patterns [42]. Similar results were also demonstrated in oral squamous cell and colon cancers [50,51]. Sankpal et al. demonstrated that p53, which is frequently mutated and inactivated in cancer, binds the EpCAM promoter at p53 candidate binding sites and represses transcription in breast cancer cell lines [52]. Wildtype p53 also silenced EpCAM expression by inducing gene methylation changes [53]. In liver cancer, histone lysine demethylase 4D (JMJD2D) reduced histone methylation levels and increased EpCAM and cancer stem marker SOX9 expression [54]. The precise role epigenetic changes play in modulating EpCAM expression during EMT remains to be clarified.

## 4. Regulated Intramembrane Proteolysis of EpCAM and EpICD Signaling during EMT

**Figure 3:**
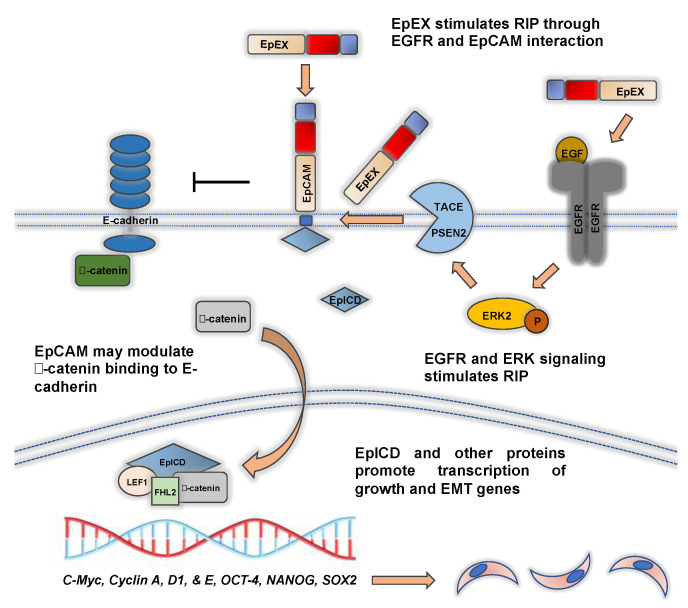
Regulated intramembrane proteolysis of EpCAM modulates EMT. EpEX and EGF simulate RIP, which is mediated by TACE and PSEN2 enzymatic cleavage. This occurs, in part, through ERK signaling. Upon EpCAM cleavage, EpICD is transported into the nucleus. EpICD binds FHL2, LEF1, and β-catenin to form a nuclear DNA binding complex that promotes transcription of target genes and in turn causes tumor growth and EMT. EpCAM has also been shown to be a negative regulator of E-cadherin. This may free β-catenin for downstream signaling with EpICD.

In 2009, it was shown that EpCAM can function as an intracellular signaling molecule and this likely plays an important role in EMT (Figure 3). EpCAM is cleaved at the cellular membrane in a process called regulated intramembrane proteolysis (RIP) by tumor necrosis factor-α-converting enzyme (TACE, also known as ADAM metallopeptidase 17 [ADAM17]) and presenilin 2 (PSEN2), which is part of the γ-secretase complex. This cleavage is stimulated by soluble EpEX binding of EpCAM, cell–cell contact, and EGFR signaling [12,55,56]. The role of EGFR signaling is discussed in further detail below. EpCAM cleavage releases the intracellular domain of EpCAM (EpICD) into the cytoplasm where it is transported into the nucleus. Nuclear EpICD binds Four-and-a-half LIM Domains 2 (FHL2), Lymphoid Enhancer Binder Factor 1 (LEF1), and β-catenin to form a DNA binding complex that stimulates the transcription of target genes. This in turn promotes tumor growth and EMT [12,44,55,56,57,58]. Upregulated nuclear β-catenin signaling is a major driver in many cancers and promotes EMT [48]. These findings suggest that RIP of EpCAM, which would presumably relieve its adhesive function, also likely promotes EMT. A recent study demonstrated that RIP and EpICD generation occurs rather slowly compared to other signaling mechanisms. The authors of this study concluded that EpICD generation by RIP is responsible for steady signaling events, as opposed to rapid transmission of extracellular cues into de novo gene transcription [59]. This would suggest that EpICD signaling may not be an initiating or driver event in EMT, but rather may play a supporting role.

In addition to its signaling role, β-catenin also binds E-cadherin at the cell surface and participates in the formation of cell adhesions. By a series of complex phosphorylation and other events, β-catenin is released from cadherins to participate in down-stream signaling [60]. Whether EpCAM directly causes the release of β-catenin from E-cadherin is unknown, but it has been shown that silencing of EpCAM in breast cancer cell lines increased β-catenin binding to the cytoskeleton [61]. Thus, by its effect through nuclear signaling (EpICD) and its negative regulation of E-cadherin (discussed below), EpCAM may be able to regulate β-catenin signaling and EMT progression through two separate mechanisms, though further research is needed. Interestingly, other studies have also shown that increased β-catenin signaling also stimulates EpCAM expression. β-catenin formed a complex with TCF4, and Tcf elements in the EpCAM promoter could bind TCF4, which in turn stimulated EpCAM gene expression [49]. Shi et al. demonstrated that cytokeratin 18 (CK18) silencing in breast cancer cells, which is observed during EMT, promoted β-catenin signaling and increased expression of EpCAM [62]. Similarly, in an in vitro and in vivo analysis of triple negative breast cancers (TNBC), reversal of EMT progression by DNA methyltransferase and histone deacetylase inhibitor treatment, decreased EpCAM cleavage and nuclear signaling with TCF4 [63].

Analysis of human tumor samples has shown that increased nuclear expression of EpICD is associated with aggressive cancer behavior, which is consistent with EpICD’s possible role in promoting EMT through β-catenin signaling. Nuclear expression of EpICD is a marker of more aggressive behavior in oral squamous cell carcinoma [64], thyroid cancer [65], breast cancer [66], cholangiocarcinoma [67], and pancreatic adenocarcinoma [68]. A comprehensive study that looked at ten epithelial cancer types demonstrated that cytoplasmic and nuclear expression of EpICD is frequently observed in cancers, but not typically observed in normal tissues. In some cancer types, membranous EpCAM expression is lost, while increased cytoplasmic and nuclear expression is present, suggesting that RIP of EpCAM has occurred [69]. In some studies, however, nuclear expression of EpICD represented a favorable prognostic marker [70], highlighting the context dependent role of EpCAM in tumorigenesis and EMT.

In summary, these studies demonstrate that EpCAM is regulated by intricate feedback loops during EMT. In a double-negative feedback loop (Figure 2), ERK silences EpCAM expression and promotes EMT. Likewise, EpCAM can inhibit ERK activity and promote an epithelial phenotype [37]. RIP of EpCAM and downstream EpICD signaling promotes growth and EMT (Figure 3) and is regulated through a positive-feedback loop. RIP releases EpEX from the cell surface and EpEX has been shown to stimulate RIP and EpICD signaling [12,55]. Increased β-catenin signaling can also increase EpCAM expression [49].

## 5. EpCAM Is Regulated by EGFR Signaling during EMT and EpEX Is an EGFR Ligand that Affects EMT

EpCAM and EMT have been shown to be regulated by EGFR signaling (Figure 3). Analysis in endometrial and colon cancer cell lines demonstrated that EGF treatment caused EpCAM cleavage by γ-secretase. In turn, EpICD, together with LEF1, promoted transcription of genes involved in EMT, including those that play a key role in junctional protein complex formation and cell migration. EGF treatment also caused increased softness, decreased adhesiveness, and increased invasion, phenotype changes consistent with EMT [44,56,71].

Several groups have shown that the extracellular domain of EpCAM (EpEX) can also serve as an EGFR ligand. Pan et al. demonstrated that EpEX binds EGFR in head and neck cancer cell lines. Unlike the findings observed in endometrial cancers, EGF treatment had minimal effects on RIP of EpCAM. Rather, EpEX functioned as a competitive EGFR ligand. Co-stimulation of EGF with EpEX blunted the effects of EGF induced EMT phenotype changes and expression of EMT transcription factors (SNAI1 SNAI2, and ZEB1). EpEX’s proliferative effects on EGFR stimulation was modulated by ERK activity [13]. In colon cancer, EpEX stimulation of EGFR promoted proliferation, migration, and stemness characteristics. This occurred via ERK signaling as well. An EpEX-EGFR-ERK signaling axis also promoted RIP and EpICD nuclear translocation. Increased nuclear EpICD expression in colon cancer specimens was associated with a worse prognosis [56,58].

Recently, EpEX-EGFR signaling was shown to promote PD-L1 expression in colon and lung cancer cell lines. This suggests that EpEX may play a significant role in cancer cell evasion of the host immunity [72], which is frequently observed with EMT [73]. In an in vitro analysis of bone marrow derived mesenchymal stem cells, EpEX treatment stimulated elaboration of stemness markers, and this occurred, in part, through EGFR signaling [74]. Together, these studies demonstrate that EpEX can negatively or positively regulate EMT through EGFR signaling. EpEX-EGFR-ERK signaling can also induce RIP of EpCAM and release of EpEX, suggesting the presence of a possible positive feedback loop involving EGFR.

## 6. EpCAM Mediates Migration and Invasion through Various Signaling Pathways and Mechanisms

While EpCAM has typically been shown to be downregulated with EMT, EpCAM can promote invasion in epithelial cancers, a phenotype typically acquired with EMT. Multiple oncogenic pathways and cellular mechanisms have been associated with this role. Notable reports are listed in Table 1, and are reviewed here. In some of these studies, the precise mechanism by which EpCAM stimulates invasion is not clearly delineated. For a comprehensive analysis, please refer to the review by Fagotto et al. [33].

In breast cancer, multiple studies suggest that EpCAM promotes invasion. In one of the first studies to characterize this phenomenon, EpCAM knockdown in MDA-MB-231 cells was shown to modulate cadherin and catenin binding to the cytoskeleton and inhibit migration and invasion [61]. The transcription factor activator protein 1 (AP-1) plays a central role in promoting cancer cell metastases and coordinating the activation and silencing of genes involved in cancer invasion. AP-1 is a dimeric protein complex consisting of Fos and Jun members [90]. EpCAM was shown to stimulate AP-1 transcription factor activity in breast cancer cell lines. EpCAM overexpression caused AP-1 activation through c-Jun subunit phosphorylation and MEK1/MKK7/JNK transduction pathway signaling. In turn, this modulated AP-1 target gene expression and promoted cancer cell invasion [79]. TGF-β1 treatment induced EMT and migration in breast cancer cell line MCF-7, and this was shown to be modulated through a JNK/AP-1/EpCAM signaling axis [80]. EpCAM has also been shown to modulate IL-8 and NF-κβ expression and invasion in breast cancer cell lines [81]. EpCAM overexpression promoted EMT and expression of stem cell markers (NANOG, SOX2, and OCT4) under hypoxic conditions in breast cancer cell lines and this effect also occurred through NF-κβ signaling as well [82]. It is important to note that EpCAM’s effect can be dependent on the cell type. In breast cancer cell lines with an epithelial phenotype, EpCAM promoted invasion, while in cell lines with a mesenchymal phenotype, EpCAM had minimal impact on invasion [83].

EpCAM has been shown to promote invasion in other cell types and cancers. In vivo experiments in mice that expressed EpCAM-deficient Langerhans cells (LC) showed decreased LC migration. It was postulated this occurred because of EpCAM’s ability to negatively regulate LC adhesions to keratinocytes, though specific functional data was lacking [87]. In nasopharyngeal cancer, EpCAM was shown to promote EMT and metastasis formation in vitro and in vivo, which was mediated by PTEN/AKT/mTOR signaling [75]. In lung cancer, increased EpCAM expression along with Metastasis-associated protein 1 (MTA1) promoted migration [77]. Oncogenic KRAS and p53 mutations in a prostate cancer mouse model caused EpCAM overexpression, which was associated with increased invasion and stemness characteristics [86]. Embryonic analysis also demonstrated that EpCAM is a novel protein kinase C (nPKC) inhibitor and regulates myosin activity. The cytoplasmic tail of EpCAM inhibited nPKC and EpCAM knockdown caused increased nPKC and ERK activity, which resulted in aberrant myosin contractility and migration [88,89]. EpCAM’s regulation of myosin activity and cell adhesions (discussed in greater detail below) and EpCAM’s signaling through EpICD (discussed above) provides a mechanistic explanation for EpCAM’s ability to modulate and promote migration and invasion.

It is also important to note that other studies have shown that EpCAM negatively affects migration and invasion or is downregulated with migration and invasion. This negative role was usually mitigated through EGFR and ERK signaling and was also typically observed in mesenchymal cell types of multiple different cancer types [13,37,44,76,78,84,85]. This is consistent with previous findings demonstrating that EpCAM negatively regulates ERK and EMT in a double-negative feedback loop [37].

## 7. EpCAM Regulates the Formation of Epithelial Junctional Protein Complexes and May Affect EMT

In addition to its homophilic adhesive properties, EpCAM has been shown to modulate other junctional protein complexes (e.g., cadherins and tight junctions) that are important for maintaining epithelial integrity and are modulated during EMT [24]. EpCAM’s impact on cadherins and tight junctions provides one possible mechanistic explanation for EpCAM’s pro-invasive effect, though further research is needed. E-cadherin and N-cadherin play essential roles in EMT. During EMT, epithelial cells replace E-cadherin expression with N-cadherin expression. This causes transitioning cells to acquire a mesenchymal phenotype and promotes cell migration and invasion [91,92]. Inducible overexpression of EpCAM was shown in E-cadherin and N-cadherin expressing cell lines to reduce aggregation caused by both cadherin types. Moreover, forced EpCAM overexpression redistributed N-cadherins from cadherin junctions to diffuse points along the cell membrane. This negative effect on cadherin adhesion activity was also associated with decreased cadherin binding of α-catenin and β-catenin, which are important linkers between cadherins and the cytoskeleton [93]. While EpCAM was shown to negatively affect both E- and N-cadherin, it is important to note that EpCAM is frequently expressed at high levels in epithelial cancers that predominantly express E-cadherin. A follow-up analysis by the same group demonstrated that EpCAM decreased E-cadherin’s intercellular adhesion strength, in part, by disrupting the binding of E-cadherin to the cytoskeleton via α-catenin and possibly through pp120 interaction as well. Interestingly, EpCAM overexpression also caused formation of actin stress fibers, which is observed during EMT [94]. In breast cancer, EpCAM is highly overexpressed in primary and metastatic lesions. Silencing of EpCAM in vitro increased cytoskeleton associated expression of E-cadherin, α-catenin, and β-catenin and decreased cell proliferation, migration, and invasion in MDA-MB-231 cells [61]. In contrast, embryological analysis in zebrafish demonstrated that EpCAM worked cooperatively with E-cadherin. Mutant EpCAM was associated with decreased E-cadherin expression [95].

EpCAM has also been shown to bind claudins and affect tight junction formation. Tight junctions are important for maintaining normal epithelial integrity at apical membranes and loss of tight junctions is associated with EMT and tumorigenesis in multiple cancer types including brain [96], breast [97], and pancreas [98] cancers. Ladwein et al. showed that EpCAM binds claudin-7 in rat and human pancreas cancer cell lines and in human tumor samples [99]. In similar studies, EpCAM was shown to bind claudin-7 and claudin-1 in human colorectal cell lines and silencing of EpCAM increased tight junction formation. Furthermore, EpCAM was upregulated in human colorectal cancers and formed a complex with claudin-7 in primary tumors and metastases. This complex formation was associated with chemotherapy resistance and decreased survival [100,101]. In HEK293 and rat pancreatic cancer cell lines, EpCAM and claudin-7 were shown to form a complex which promoted migration and tumor growth, and this was associated with increased ERK signaling. Binding of claudin-7 occurred through the TM domain of EpCAM, and also prevented EpCAM oligomerization [102]. Interestingly, it was also demonstrated that claudin-7 associated TACE and PSEN2 cleaved EpCAM, which subsequently increased EpICD signaling and expression of EMT transcription factors [103].

Together, these studies demonstrate that EpCAM modulates junctional protein complex formation and function and regulates epithelial adhesions formed by cadherins and claudins. Claudins can also affect RIP and EpICD signaling. While EpCAM itself can form homophilic adhesions (albeit weaker than E-cadherin), its effect can also possibly reduce overall adhesion strength by affecting other adhesion molecules. Loss of cellular adhesion strength and commensurate relief of contact inhibition is a major feature of EMT and cancer initiation [24,104]. In cancer, this may be controlled somewhat paradoxically by EpCAM’s selective and relatively weak aggregative abilities in conjunction with its negative effects on cadherin and tight junction formation. That said, it remains to be determined if EpCAM’s effect on cadherins and claudins drives EMT.

## 8. EpCAM Expression in Epithelial and Mesenchymal Cancers

Analysis of EpCAM’s expression patterns in diverse cancer specimen types has provided important insights into EpCAM’s role in EMT. In general, tumors of mesenchymal origin have low EpCAM expression, while cancers of epithelial origin have high EpCAM expression. Accordingly, low EpCAM expression was observed in melanoma, some neurological cancers, lymphomas, and sarcomas [15], while high EpCAM expression was observed in breast [61], gallbladder [105], gastric [106], pancreatic [107], prostate [108,109], and colorectal cancers [110]. Some epithelial cancers, however, demonstrated low EpCAM expression, including squamous cell cancers, hepatocellular carcinomas, clear cell renal carcinoma, and urothelial cancers [111], while some sarcomas have shown elevated EpCAM expression [112]. EpCAM expression is a negative prognostic marker in most epithelial cancers, though exceptions are noted. For a comprehensive analysis of EpCAM’s prognostic significance in cancer, please refer to the review by van der Gun et al. [34]. EpICD participates in nuclear signaling and has a critical role in regulating EMT (discussed above). It is important to note that many studies do not clearly distinguish the cellular location of EpCAM expression in these analyses (e.g., membranous, cytoplasmic, or nuclear).

In a comprehensive study that examined EpCAM expression in primary and solid metastatic lesions, high EpCAM expression was frequently present in metastases of epithelial cancers. The expression patterns in the metastases generally mirrored the expression patterns in the primary tumor, indicating that EpCAM expression is important for metastatic development in epithelial cancers [111]. Indeed, in breast and prostate cancers, higher EpCAM expression has been observed in metastatic lesions compared to the primary tumors [109,113].

In some epithelial cancers, EpCAM expression varies within tumor subtypes and representative examples are discussed here. EpCAM is frequently overexpressed in ductal and metastatic breast cancers and high EpCAM expression can serve as a poor prognostic marker [61,114,115,116]. Lobular and other histological breast cancer subtypes, however, usually have lower EpCAM expression [111,115,116]. Pathological changes associated with EMT have not usually been observed in lobular breast cancer [117] and it remains uncertain what is the cause of lower EpCAM expression in this breast cancer subtype. Triple negative breast cancer (TNBC) is an aggressive form of breast cancer that expresses a mesenchymal phenotype [118], but also retains EpCAM expression. Indeed, a recent analysis of TNBC specimens demonstrated that EpCAM and EMT markers were frequently co-expressed and this expression pattern was associated with lymph node metastases and decreased survival [119]. Similar results were also shown by Soysal et al. in their analysis of basal-like breast cancer [116].

Well differentiated thyroid cancer, including papillary and follicular thyroid cancer, has high EpCAM expression. EpCAM expression is progressively lost with tumor dedifferentiation in poorly differentiated (PDTC) and anaplastic thyroid (ATC) cancers. Interestingly, membranous EpEX expression was associated with improved survival, while nuclear EpICD expression was associated with decreased survival [65,120]. PDTC and ATC have high expression of EMT markers and EMT is an important driver in thyroid cancer dedifferentiation and progression [121,122]. This suggests that loss of EpCAM expression in aggressive thyroid cancers is related to EMT.

Together, these tissue studies demonstrate that EpCAM promotes tumorigenesis in epithelial cancers, but likely has a minimal role in mesenchymal cancers. Indeed, in a comprehensive analysis that examined EMT gene expression signatures in 166 cancer cell lines and in breast and lung cancers, EpCAM expression was significantly less or absent in mesenchymal-like cancer cell lines and tumors [37].

## 9. EpCAM, Circulating Tumor Cells, and EMT

During the early steps of epithelial metastasis, cancer cells undergo EMT to detach from the primary tumor, invade the surrounding tissues, and disseminate hematogenously as circulating tumor cells (CTCs). CTCs are detectable in human blood samples of cancer patients, generate metastases, and contribute to cancer progression. Detection of elevated CTCs in clinical blood samples is typically associated with a worse prognosis and CTCs levels and types can be monitored to determine the response to therapeutic interventions. EpCAM is frequently used as a target to capture and measure CTCs [123,124,125]. As an epithelial marker, EpCAM has successfully been used in the CellSearch system [22] and others to capture and measure CTCs. Detection of EpCAM positive CTCs has provided prognostic information in breast [126], liver [127], prostate [128], colorectal cancer [129], and neuroendocrine cancer patients [130]. These findings demonstrate that EpCAM supports CTC propagation and the metastatic cascade in epithelial cancers.

Multiple studies demonstrate that EpCAM expression is retained in CTCs that are affected by EMT. This is consistent with emerging evidence showing that cancer cells which express an intermediary EMT phenotype may have increased metastatic potential [131]. Liu et al. studied the effects of EpCAM and EMT in CTCs and DTCs in a mouse model of metastatic breast cancer. Of the different types of CTCs identified, CTCs with a moderate mesenchymal transition that retained EpCAM expression had greater metastatic potential compared to CTCs with a prominent mesenchymal phenotype without EpCAM expression [132]. Analogous results were also demonstrated in a mouse model of prostate cancer [133]. EpCAM positive CTCs that co-express EMT markers were frequently detected in breast and prostate cancer patients [134,135]. Similarly, detection of EpCAM positive CTCs with high expression of EMT markers (ETV5, NOTCH1, SNAI1, TGFB1, ZEB1, and ZEB2) was associated with a worse prognosis in endometrial cancer patients [136]. CTCs that are isolated and cultured from epithelial cancer patients frequently retain EpCAM expression, but also express mesenchymal markers [137,138,139]. Together, these results demonstrate that CTCs expressing EpCAM and an intermediary mesenchymal phenotype are an important driver of metastatic formation in epithelial cancers.

It has also been shown, however, that CTCs can undergo EMT and downregulate EpCAM expression [140,141]. Indeed, in an important study, Yu et al. showed that breast CTCs acquire an increasingly mesenchymal phenotype with treatment resistance and cancer progression [118]. Jojovic et al. showed in 1998 that newly formed, small metastatic foci in a mouse model of colon cancer lacked EpCAM expression, while EpCAM expression was observed in larger metastatic lesions. The authors reasoned that EpCAM expression was silenced during the early stages of tumor cell dissemination and metastasis formation, and that this was related to EMT [142]. Similar results were also shown in an analysis of human cases of metastatic cancer by Rao et al. The authors demonstrated that the expression of EpCAM on CTCs was approximately 10-times lower compared to primary and metastatic lesions [143]. In esophageal cancer patients, EpCAM expression was lower in lymph node and bone marrow disseminated tumor cells (DTCs). EpCAM silencing by short-hairpin RNA (shRNA) in esophageal squamous cancer cell lines induced EMT changes and promoted increased metastatic formation. Interestingly, EpCAM expression was frequently reconstituted in the metastatic lesion formed by the shRNA EpCAM knock-down clones [76]. Similar results were also observed in a mouse model of metastatic prostate cancer using DU145 cells. Progressive rounds of metastatic formation in mouse lymph nodes by DU145 cells initiated mesothelial-to-epithelial transition (MET) and increased epithelial marker expression, including EpCAM [144]. These results show that EpCAM is dynamically regulated during metastatic formation with changes in EpCAM expression patterns during EMT and MET.

Accordingly, multiple studies have shown that loss of EpCAM expression with EMT in CTCs is associated with increase metastatic potential and worse prognosis. Tachtsidis et al. examined xenograft models of triple negative (using MDA-MB-468 cell line) and ER-positive (patient derived) breast cancer and showed that low EpCAM and high EMT marker expression in CTCs promoted tumor metastases [145]. Neoadjuvant and salvage chemotherapy increased EpCAM negative, EMT positive CTCs in breast cancer patients, suggesting that the loss of EpCAM expression in CTCs may enhance chemotherapy resistance [118,146]. In pancreatic cancer, detection of CTCs with mesenchymal markers and minimal EpCAM expression portended a worse prognosis and was associated with higher stage tumors and metastatic disease [147]. Similar results were shown in an in vivo model of hepatocellular carcinoma [148].

## 10. EpCAM, Cancer Stem Cells, and EMT

EpCAM and EpICD can also regulate and/or be controlled by reprogramming factors involved in promoting tumor initiating cells (TICs), also known has cancer stem cells (CSC). CSCs represent a subpopulation of tumor cells that have embryonic and mesenchymal properties. CSCs supply a self-renewing source of cancer cells and promote cellular dedifferentiation and tumor growth. It is believed that CSCs frequently form during EMT, and CSCs share many of the same markers and phenotypic properties that characterize EMT [149]. In normal human epithelial stem cells, EpCAM was shown to be expressed in OCT4 positive embryonic stem cells and EpCAM silencing had inhibitory effects on growth and stemness characteristics [150]. In colon cancer, EpCAM served as a CSC marker and was shown to be upregulated during signaling that promotes EMT [151,152]. Increased EpCAM expression occurred with other CSC markers in chemotherapy resistant LoVo colon cancer cells, which was mediated by CXC ligand signaling [153]. EpICD was shown to stimulate the transcription of CSC reprogramming genes, including OCT4, NANOG, c-MYC, and SOX2, and promoted self-renewal properties [58,154]. Similar results were also observed in kidney and fibroblast human cell lines [155].

EpCAM is not typically expressed in normal hepatocytes, but EpCAM expression has been observed in embryonic liver tissue and in regenerative stem cells of the adult liver [156]. In hepatocellular carcinoma (HCC), EpCAM is considered a CSC marker [157] and EpCAM positive HCC cell lines were shown to have increased proliferative properties compared to EpCAM negative cells [158]. Tumor associated macrophages and hepatic stellate cells are part of the tumor micro-environment and support HCC development. Both cell types have been shown to stimulate EpCAM expression, EMT/stemness characteristics, proliferation, and migration. This was mediated, in part, through TGB-β1 signaling, a prominent inducer of EMT [159,160]. EpCAM is frequently co-expressed with EMT and stemness markers in human HCC specimens and positive EpCAM expression has been associated with a worse prognosis [159,161]. EpCAM expression, however, has also been shown to be negatively associated with other important CSC/EMT characteristics in HCC, including chemotherapy and molecular therapy resistance [158,162]. Thus, it seems that EpCAM expression may be necessary to promote and maintain some CSC and EMT properties, but is unnecessary for other features.

EpCAM is expressed in fetal pancreatic tissue [163] and EpCAM+/CD24+/CD44+ pancreatic adenocarcinoma cells were identified as CSCs with potent tumorigenic potential [164]. Notably, these CSCs also expressed epithelial (E-cadherin) and mesenchymal (N-cadherin, Vimentin, and SNAI2) markers. Inhibition of Notch signaling (a mediator of CSCs) in EpCAM+/CD44+ pancreatic CSCs decreased EpCAM and mesenchymal marker expression, and also had inhibitory effects on in vitro and in vivo growth [165].

## 11. EpCAM and Drug Resistance during EMT

Chemotherapy treatment and molecular therapies frequently affect EMT, stemness characteristics, and EpCAM expression. Cisplatin treatment of primary human ovarian cancer cells and the ovarian cancer cell line OVCA 433 induced EMT and CSC marker overexpression, which also included increased EpCAM expression. The observed changes were controlled, in part, by ERK2 signaling, but it was not specifically tested whether changes in EpCAM expression were caused by ERK2 signaling [166]. Another study that analyzed CTCs in ovarian cancer patients similarly showed that chemotherapy induced EMT changes, but EpCAM expression decreased in tested CTCs [167]. In breast cancer, loss of EpCAM expression in CTCs served as a marker of chemotherapy resistance [146], while another study showed increased EpCAM expression in treatment resistant patients [168].

## 12. Future Directions

Crystal structural analysis has demonstrated that the second motif of EpCAM between amino acids 63 and 135 resembles a type 1 thyroglobulin domain (TY-1) [25]. TY-1 domains are present in many other proteins and can function as cathepsins-L (CTSL) inhibitors [169]. It has been hypothesized that EpCAM may be able to inhibit CTSL activity as well [1]. CTSL is highly expressed in many cancers [170,171], degrades extracellular matrix (ECM) proteins, and promotes cancer cell invasion and metastases [172]. Preclinical studies have demonstrated that CTSL targeting in cancer patients may be an effective therapeutic strategy [173]. The ectodomain of EpCAM and CTSL are both secreted into the tumor micro-environment of cancer patients [20,174,175,176], whereby EpCAM could possibly inhibit CTSL activity. This would presumably have a negative effect on EMT progression, as the ability to degrade the ECM is a hallmark feature of EMT progression. We recently demonstrated that the TY-1 domain of EpCAM can inhibit CTSL activity [177].

## 13. Conclusions

EpCAM has a complex and sometimes contradictory role during EMT and is an important area for future research. EpCAM is an epithelial marker and is expressed on the basolateral membrane of normal epithelial cells, promotes cell adhesions, and plays an important role in maintaining the epithelial integrity of the gastrointestinal tract. EpCAM is downregulated with EMT and inhibits EMT in a double-negative feedback loop that involves ERK signaling. EMT transcription factors frequently silence EpCAM expression. In head and neck cancers, EpEX stimulation of EGFR had negative effects on EMT progression, though it promoted cell growth. Many studies have demonstrated that EpCAM expression was inversely related to the expression of mesenchymal markers and EMT transcription factors. Most mesenchymal tissues and cancers have minimal or no detectable EpCAM expression, while high EpCAM expression is frequently present in epithelial cancers. Many cancer types lose EpCAM expression during tumor cell dissemination with EMT and/or dedifferentiation.

As outlined above, EpCAM may be able to promote EMT by negatively regulating epithelial junctional protein complex formations. This occurs, in part, through direct interactions with junctional proteins and possibly through indirect signaling mechanisms. EpICD functions as a nuclear signaling molecule and promotes EMT progression through β-catenin pathway activation. Many studies have shown that EpCAM and EpICD expression promotes cancer cell migration and invasion, a phenotype typically associated with EMT. Analysis of human tumor samples demonstrate that nuclear EpICD expression is associated with more aggressive cancer behavior and decreased survival. EpCAM expressing CTCs that also elaborate mesenchymal markers promote metastasis formation and serve as a negative prognostic maker. EpCAM has been demonstrated in clinical studies to be a promising target in the detection and treatment of metastatic epithelial cancers.

Thus, EpCAM demonstrates a dynamic and context dependent role during EMT and cancer initiation and progression. Future research exploring EpCAM’s role in EMT may reveal additional therapeutic opportunities in the diagnosis, treatment, and monitoring of patients with epithelial cancers.

## Figures and Tables

**Table 1:** EpCAM is associated with migration and invasion modulation via various signaling pathways and mechanisms.

Cell Type & Cell Line	Associated Signaling Pathway/Mechanism	Promotes/Inhibits	Reference
Head and Neck Squamous Cell Carcinoma: FaDu and Kyse-30	EpEX & EGFR	Inhibits	[13]
Nasopharyngea S Carcinoma: S18, 6-10B, and HONE1	PTEN/AKT/mTOR	Promotes	[75]
Esophageal Squmous Cell Carinoma: Kyse-30 and Kyse-520	EMT	Inhibits	[76]
Lung Cancer: A549 and NCI-H446	MTA1	Promotes	[77]
Benign Breast: MCF-10A and Human Mammary Epithelial Cells	ERK & EMT	Inhibits	[37,78]
Breast Cancer: MDA-MB-231	E-Cadherin & α,β-catenin	Promotes	[61]
Breast Cancer: MDA-231 and CA1a	AP-1	Promotes	[79]
Breast Cancer: MCF-7	TGF-β1	Promotes	[80]
Breast Cancer: MDA-MB-231	NF-κβ	Promotes	[81,82]
Breast Cancer: MCF-7, T47D, SkBR3, MDA-MB-231, and Hs578t	EMT	Both	[83]
Kideny: MDCK	ERK, Claudin-7, & actomyosin contractility	Inhibits	[84]
Colon: HCT116	EpEX & EpICD	Promotes	[55]
Endometrial: Ishikawa and RL95-2	Adhesion formation & EpICD	Both	[44]
Ovarian: SKOV3 & OVCAR4	ERK	Inhibits	[85]
Prostate: Kras^G12D^ & p53^L/L^ mouse knockout prostate cancer model	KRAS & p53	Promotes	[86]
Langerhans cells: Knockout mice model	Decrease adhesiveness	Promotes	[87]
Embryonic and Colon: Xenopus laevis and Caco-2 and SW480	nPKC and myosin regulation	Promotes	[88,89]

## Data Availability

Not applicable.

## Abbreviations

ADAM17	ADAM metallopeptidase 17
AP-1	activator protein 1
ATC	anaplastic thyroid cancer
BAA	bead aggregation assays
CAM	cell adhesion molecule
CD	carboxyl-terminal domain
CSC	cancer stem cells
CTC	circulating tumor cells
CTE	congenital tufting enteropathy
CTSL	cathepsins L
DTCs	disseminated tumor cells
ECM	extracellular matrix
EGFR	epidermal growth factor receptor
ERK	extracellular signal-regulated kinases
EpCAM	epithelial cell adhesion molecular
EpEX	EpCAM extracellular domain
EpICD	EpCAM intracellular domain
FHL2	four-and-a-half LIM domains 2
FILM-FRET	fluorescence-lifetime imaging based Förster resonance energy transfer
EMT	epithelial-to-mesenchymal transition
JMJD2D	histone lysine demethylase 4D
kb	kilobases
kD	kilodalton
LC	Langerhans cells
LEF1	lymphoid enhancer binder factor 1
MET	mesenchymal-to-epithelial transition (MET)
MMPs	matrix metallopeptidases
ND	N-terminal domain
nPKC	novel protein kinase C
OCT4	octamer-binding transcription factor 4
PDTC	poorly differentiated thyroid cancer
PSEN2	presenilin 2
RIP	regulated intramembrane proteolysis
shRNA	short-hairpin RNA
SAXS	small angle X-ray scattering
SP	signal peptide
TACE	tumor necrosis factor-α-converting enzyme
TCF4	transcription factor 4
TGF-β1	transforming growth factor- β1
TNBC	triple negative breast cancer
TM	transmembrane
TY-1	type-1 thyroglobulin
ZO-1	zona occludens-1
